# Notch Signaling in Breast Tumor Microenvironment as Mediator of Drug Resistance

**DOI:** 10.3390/ijms23116296

**Published:** 2022-06-04

**Authors:** Adele Chimento, Maria D’Amico, Vincenzo Pezzi, Francesca De Amicis

**Affiliations:** 1 Department of Pharmacy and Health and Nutritional Sciences, University of Calabria, 87036 Arcavacata di Rende, CS, Italy; adele.chimento@unical.it (A.C.); dmcmra96b48d086r@studenti.unical.it (M.D.); francesca.deamicis@unical.it (F.D.A.); 2 Health Center, University of Calabria, 87036 Arcavacata di Rende, CS, Italy

**Keywords:** JAG, RBPJ, γ-secretase, tumor-associated macrophages, cancer-associated fibroblasts

## Abstract

Notch signaling dysregulation encourages breast cancer progression through different mechanisms such as stem cell maintenance, cell proliferation and migration/invasion. Furthermore, Notch is a crucial driver regulating juxtracrine and paracrine communications between tumor and stroma. The complex interplay between the abnormal Notch pathway orchestrating the activation of other signals and cellular heterogeneity contribute towards remodeling of the tumor microenvironment. These changes, together with tumor evolution and treatment pressure, drive breast cancer drug resistance. Preclinical studies have shown that targeting the Notch pathway can prevent or reverse resistance, reducing or eliminating breast cancer stem cells. In the present review, we will summarize the current scientific evidence that highlights the involvement of Notch activation within the breast tumor microenvironment, angiogenesis, extracellular matrix remodeling, and tumor/stroma/immune system interplay and its involvement in mechanisms of therapy resistance.

## 1. Introduction

Breast cancer (BC) is the most recurrently diagnosed cancer in women and the incidence rate of such neoplasia is higher in developed countries and varies significantly according to race and ethnicity. It represents a very heterogeneous group of neoplasms for morphology, prognosis and response to therapy [1]. It is generally classified according to clinical and pathological characteristics: age, tumor size, involvement of axillary nodules, angiolymphatic invasion, histological degree and hormonal receptors status [1]. Particularly, ERα, PR and HER2 exert different effects in vitro and in vivo [2,3,4,5]. Clinically based on immunohistochemical analysis, BC is divided into three subtypes: ERα+ and/or PR+, HER2+ and TNBCs that lack expression of ERα, PR, and HER2.

In addition to traditional subtype analysis, BC can also be subtyped based on gene expression profiles [6]. Five subtypes (luminal A, luminal B, basal-like, normal-like, and HER2-like), each characterized by specific gene expression profiling, were identified by microarray gene expression studies on a series of invasive mammary tumors followed by a hierarchical grouping of differentially expressed genes [6].

Several studies have implemented these acquisitions and evidenced that three gene sets are particularly important for BC prognosis [7,8,9]. The first gene set is the “proliferation metagenes”, which is associated with worse prognosis, particularly in ER+ carcinomas [9,10]. The second refers to the immune cell-associated “B-cell and T-cell metagenes” that are associated with better prognosis, particularly in fast proliferating carcinomas [9,11]. The third pattern, the “ER associated metagenes”, is of limited prognostic relevance in node-negative BC but is important when dissecting tumors according to biological processes [9,10]. Thus, the phenotypic diversity of BC is related to gene expression pattern diversity. Interpretation of their patterns of variation will lead to efficient targeted therapies for BC patients, even though the definition of novel signals driving BC progression and drug resistance is urgently needed to improve the clinical outcome.

Numerous studies confirm that the Notch pathway has a major participation in BC progression and therapy resistance [12]. Notch activation is a hallmark of the TNBC [13] and contributes to the pathogenesis of human BC by affecting multiple cellular processes, including cancer stem cell maintenance, cell fate specification and differentiation [14]. Recent evidence suggests that Notch is involved in tumor morphogenesis and patterning of the tumor microenvironment (TME) [15].

## 2. The Notch Signaling Pathway

**Figure 1:**
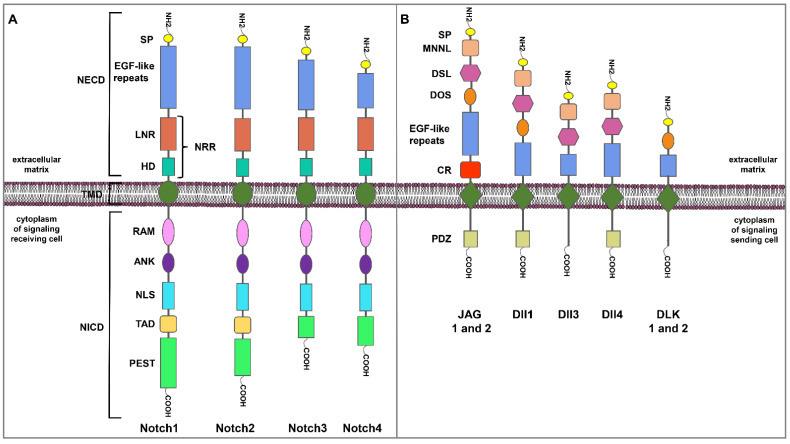
Schematic representation of human Notch receptors and ligands families. (**A**) Notch receptors comprise an extracellular domain (NECD), a transmembrane domain (TMD), and an intracellular domain (NICD). The NECD contains signal peptide (SP) sequence, multiple epidermal growth factor (EGF)-like tandem repeats, Lin12-Notch repeats (LNR) and a heterodimerization domain (HD). The negative regulatory region (NRR) is formed by LNR and HD. The NICD contains RBPJκ-association module (RAM), nuclear localization sequence (NLS), Ankyrin (ANK) repeats, and a transcriptional activator domain (TAD), which is followed by proline (P)-, glutamic acid (E)-, serine (S)- and threonine (T)-rich (PEST) sequence. (**B**) There are five canonical Notch ligands within two families, Jagged (JAG1 and 2) and DLL family (Dll1, 3, 4), according to the length and subtype of EGF-like repeats. Additional non-canonical Notch ligands are DLK1 and 2. All canonical Notch ligands are transmembrane proteins that share a similar structure: an extracellular domain including multiple EGF repeats, cysteine-rich Domain (CR) (in the JAG1 and 2), delta and OSM-11-like Proteins Domain (DOS) (in the JAG1 and 2, Dll1), Delta/Serrate/Lag-2 (DSL), N-terminus of Notch ligands (MNNL) domain and an SP sequence; the intracellular domain of JAG 1 and 2, and Dll1 and Dll4 also include PDZ motif.

Different Notch receptors (Notch1 to Notch4) (Figure 1A) and canonical activators of the core Notch pathway, the Delta/Serrate/Lag-2 (DSL) ligands Jagged (JAG) 1 and 2, and Delta-like ligand 1 (Dll1), 3 (Dll3) and 4 (Dll4) have been defined; moreover, non-canonical Notch ligands Delta-like 1 and 2 homolog (DLK 1 and 2) are known (Figure 1B) [16,17].

Notch receptors are composed of N-terminal EGF repeats, located at the extracellular portion of Notch receptor transmembrane proteins [16]; a negative regulatory region (NRR) at a juxtamembrane portion; a transcriptional activation domain with high binding affinity for RBPJ; six Ankyrin (ANK) repeats, with low binding affinity for RBPJ and high affinity for Deltex (DTX); and a C-terminal degron domain rich in the amino acids Proline, Glutamate, Serine, and Threonine (PEST) at the intracellular region [16]. After the interaction of specific ligands, the first proteolytic cleavage of the Notch protein is followed by a further subsequent proteolytic cleavage mediated by the γ-secretase enzyme complex [17]. Therefore, the released receptor portion, the so-called Notch Intracellular Domain (NICD), moves into the nucleus where it forms a complex with the DNA binding protein RBPJ, which recruits another co-activator, a member of the MAML family [17]. This new tri-protein complex recruits a cascade of factors to drive transcription of target genes such as members of bHLH transcription factors but also cyclin D1 and Slug [18], both associated with tumorigenesis [19,20]. Glycosylation of the Notch Extracellular Domain (NECD) has emerged as an elegant means for regulating Notch activity [21], altering the affinity of the Notch receptor for DSL. The intracellular fragment of Notch3 binds with GSK3-β through the ANK repeat domain and this binding is enhanced in a proteasomal inhibition-dependent manner [22], thus influencing the stability of NICD and thereby the duration of signaling.

In addition to canonical Notch signaling, a number of non-canonical signals have been described in cancer [17]. In ER+ BC cells, Notch1 activates NF-κB- and ERα-dependent transcription [23]. In mammalian cells, NICD physically interacts with β-catenin [24], Smad proteins [25] and HIF-1α [26], thereby providing a means for direct crosstalk between Notch and the Wnt, TGF-β and hypoxia-dependent signaling pathways.

The Notch pathway influences cell fate during development and morphogenesis [27]. Notch signaling dysregulation and somatic alterations in the genes encoding Notch signaling components drive various types of human cancer [14,28,29]. Chromosome 9–7 translocation that generates a constitutively active form of Notch1 causes the Notch signaling dysregulation which is involved in T-Cell Acute Lymphoblastic Leukemia (T-ALL) [30]. Actually, genome-scale sequencing studies identified mutations in Notch genes leading to inappropriate or dysregulated activation of Notch signaling in colorectal cancer [31], glioblastoma [32], BC [12,33] and other malignancies [28]. Notch pathway stimulation causes inhibition of apoptosis, induction of proliferation and epithelial mesenchymal transition (EMT), maintenance of a stem-like phenotype, induction of angiogenesis, promotion of metastasis and drug resistance, and other tumor–stroma interactions that are less specific to individual tumor types [28]. Therefore, several cancer therapeutic strategies are designed because of systemic, pan-Notch inhibition, including the non-specific GSIs or Notch receptor-specific or Notch ligand-specific monoclonal antibodies [34,35]. These agents have not yet produced significant clinical results in early clinical trials, although they have demonstrated therapeutic activity in numerous preclinical models [35].

## 3. Notch Signaling Dysregulation in BC

High expression levels of Notch receptors and their ligands have been identified in BC tissues [36]. It was discovered that the Notch4 locus is a common integration site for MMTV and the constitutive ligand-independent activation of Notch4 leads to a greater activation of its target genes and a higher risk of onset of mammary adenocarcinoma [37,38]. Increased expression of Notch1, Notch3 [39] or Notch4 [40] is able to induce breast epithelial cell transformation into cancer cells (Table 1).

Authors suggest that the mechanism dependent on Notch1 induces changes in cell shape of the non-transformed breast cell line MCF-10A, increases cell proliferation, colony formation and resistance to apoptosis [41]. Increased proliferation and cellular invasiveness have been found in TNBC cells ectopically expressing Notch4, while inhibition of Notch4 reduces cell proliferation and tumorigenicity [42]. However, as regards the role of Notch3, it upregulates the expression of anaphase-promoting complex (APC) coactivator Cdh1 in human BCs, resulting in accumulation of CDKI p27Kip, leading to cell cycle shutdown in phase G0/G1 [48].

Studies on the expression and activation of Notch receptors in primary breast tumors suggest that Notch signaling could be a prognostic and/or predictive biomarker. Early studies have shown that normal breast tissue has high expression of the negative Notch regulator, Numb, and that its expression is lost in breast tumors [49]. Elevated Notch1 and/or JAG1 predict the poorest overall survival outcome for BC patients [36]. Interestingly, Notch2 could play a tumor-suppressive role in human BC. In particular, Notch2 expression was high in well-differentiated tumors and reduced in breast tumors with poor differentiation, while Notch1 may possess tumor-promoting functions. These results support the notion that only specific suppression of Notch1 activity may represent a novel therapeutic strategy [50].

Notch signaling can also exert oncogenic potential through its crosstalks with other signaling pathways [51] and, specifically, a crosstalk between Notch and ER signaling has been documented in BC [52]. These data show that estrogen decreases Notch signaling through an ERα-dependent effect, which is at least in part mediated by inhibition of Notch cleavage by γ-secretase. In such a way, uncleaved Notch1 accumulates in the membrane of estrogen-treated cells, indicating an effect on Notch intracellular trafficking or induction of other γ-secretase substrates that could compete with Notch1 [43]. Moreover, Notch interferes with HER2 in DCIS, suggesting that inhibition of these two pathways could be more effective in targeting BCs [44]. It is known that, in many types of cancers including breast, multiple receptor Tyrosine Kinases stimulate Ras signaling [53], and Ras overexpression/activation induces upregulation of Notch1 [45]. In this regard, Notch1 and Ras/MAPK cooperate in the transformation of immortalized breast cells. In agreement with these data, active Notch1 and active MAPK are associated with high node positivity [45], suggesting that Notch–Ras/MAPK crosstalk may lead to more aggressive breast disease. Interestingly, most samples from BC patients with Notch1 and MAPK hyperactivation included highly aggressive TNBCs that were enriched in Oct4, Nanog and CD44 stem cell markers [45]. However, we retain that the crosstalk between Notch and other signaling pathways establishes which role of Notch is implemented in a specific setting.

CSCs play an important role in the initiation and metastasis of BC [54]. It is reported that Notch1 activates the self-renewal of breast CSCs to increase the transcription of HER2 [55]. Several studies reported molecular mechanisms, indicating that Notch1 and Notch4 drive drug-resistant breast CSCs [56].

In addition, Notch ligands play a role in BC. Elevated expression of JAG1 correlates with poor overall survival in BC patients [36] and promotes angiogenesis in endothelial cells [57]. In addition, E-cadherin expression levels are inhibited as a result of Slug induction by JAG1 activation of Notch1, thus promoting EMT in human breast epithelial cells [46]. The expression of Dll1 has been associated with poor prognosis [47] and the tumor-promoting function of Dll1 is exclusive to ERα+ luminal BC, as loss of Dll1 inhibits both tumor growth and invasive potential of luminal BC [47]. Surprisingly, Dll1 expression has shown no such effect in other BC subtypes. Previously, Kontomanolis et al. observed Dll4 expression levels in plasma and neoplastic tissues of BC patients. Authors suggest that Dll4 expression is highly correlated with metastasis in BC [58]. These comprehensive studies have provided enough evidence to identify Notch as a potential therapeutic target to design effective strategies for additional novel treatment in BC.

## 4. Notch Signaling Regulates BC Progression

The current theory is that Notch, through cell cycle deregulation, inhibition of apoptosis, reprogramming of differentiation, EMT, angiogenesis and self-renewal of CSCs, promotes tumorigenesis and tumor progression [14]. In addition, it is becoming increasingly recognized that breast tumor progression depends not only upon drivers within premalignant or malignant cells, but also upon the activities of non-malignant cells that populate the TME, orchestrated by different signals including Notch.

### 4.1. The Breast TME and Disease Progression

The TME is a multifaceted environment, with dynamic cell–cell and cell–extracellular matrix (ECM) interactions crucially contributing to cancer development and progression [59,60]. The TME is classified into three distinct levels such as intratumor, regional and distant-metastatic [61] and is composed of various cell types including CAFs, MSCs, TAMs, endothelial cells, myoepithelial cells, and immune cells. In addition, it also includes components of ECM and different soluble factors [61]. Tumor development influences its microenvironment, and the microenvironment cells affect tumor growth by secreted cytokines and growth factors [60]. The crosstalk between stromal cells and immune cells induces a series of events that favor breast tumor progression [62]. The secreted soluble factors suppress immune cells or stimulate other cells to proliferate, migrate, differentiate and generate or damage ECM. This complex interaction between cells and ECM therefore leads to the formation of more invasive cancer cells that can break the connective tissue and metastasize [62]. At the same time, the bidirectional crosstalk between cancer cells and the surrounding stromal components influences the therapeutic response in BC patients [62]. Thus, strategies targeting the tumor–TME interactions could pave the way to a new generation of therapies.

Among the different cell types surrounding the tumor mass, CAFs, which are responsible for “reactive stroma”, represent the largest population of stromal cells that affect the characteristics of BC [61,63]. Secretive tumor factors regulate and control the differentiation of CAFs precursors, such as MSCs or normal tissue-resident stem cells fibroblasts, in CAFs. Furthermore, CAFs modulate ECM, secrete tumor-promoting growth factors and chemokines, which stimulate breast tumorigenicity [64]. Particularly, CAFs secrete SDF-1, which stimulate cancer cell growth via CXCR-4 receptor [65], but also different factors such as TGF-β and VEGF, IL-6, mediating stemness and EMT [66]. In addition, CAFs may also play a central role in the onset of brain metastases in BC patients [67]. Co-culturing of breast tumor cells and fibroblasts increases cancer cell growth via metabolic reprogramming of fibroblasts producing Lactase and 3-Hydroxybutyrate, a hallmark of CAFs [68]. Studies have shown that CAFs differ from fibroblasts in adjacent normal breast tissue, as they have distinct mRNA and protein expression profiles and might impact the transcriptional profile of BC cells [69,70]. It is now thought that the altered phenotype of CAFs is mainly due to epigenetic modulation of the DNA [71]. Moreover, in the TME, diverse CAF subsets interact with BCs, consequently modulating the response of tumor cells to drugs [62] and can be responsible for the resistance to chemotherapy [72]. Thus, CAFs play an active role in modeling the TME to support cancer cell survival, dissemination, angiogenesis, immune suppression and resistance to therapy.

Another important cell population in the breast cancer TME is represented by TAMs, which are characterized by a particular phenotype that induces the promotion of cancer, growth, angiogenesis, tissue remodeling and the suppression of adaptive immunity [73]. In general, macrophages can be classified as classically (M1) or alternatively (M2) activated [73]. M1 promote inflammation and constitute a major source for pro-inflammatory cytokines such as TNF-α and IL-1β [73,74]; whereas these cytokines can promote anti-tumor activities at certain stages of the malignancy process, they are also often linked to chronic inflammation and to pro-metastatic effects in different types of cancer including breast. Anti-inflammatory, M2-polarized macrophages, and most TAMs which belong to the M2 phenotype, induce cancer cells’ survival and dissemination through IL-10, CCL2, CCL17, CCL22 and TGF-β secretion [73,74]. They also block antitumor immunity by attracting T-cell subsets lacking cytotoxic functions [73,75]. In agreement with this, several studies have shown that a high population of TAMs are related to a worse prognosis of BC [76]. TAMs influence continuous matrix deposition and remodeling, allowing the invasion of the surrounding tissue [77]. Numerous studies have linked increased TAMs population to metastasis and a worse prognosis in BC, suggesting that TAMs depletion or reprogramming could represent a viable therapeutic strategy [77]. However, the relative contribution of the different macrophage phenotypes to breast cancer growth and progression and how polarized macrophages influence responses to therapy are still not clear. Further studies could be needed to demonstrate the antitumor effects of macrophages manipulating the M1/M2 balance in breast tumors.

More recently, TILs are emerging as one of the key components in the breast tumor microenvironment. High-grade DCIS are characterized by abundant TILs compared with low-grade DCIS [78]. A similar TIL pattern associates with high-grade HER2+ or TNBC subtypes. However, distant metastatic tumors show a reduced TIL population compared with matched primary tumor sites, with brain metastases having the lowest T-cell infiltration among all metastatic sites [79], suggesting that BC progression is related to an impairment in antitumor immunity. Tumors escape immune-surveillance, inhibiting the immune responses. Suppressive immune cells, chemokines and altered ECM orchestrate an antitumor immunity and, subsequently, BC progression. Therefore, novel therapeutic options, designed to modulate the immune system, increase antitumor activity acting on the TME. For instance, using combination therapy, tumors are treated with inhibitors against the primary driver, and then with drugs that work in vitro models of resistance at the same time, thus preventing recurrence. A major challenge for BC therapy is the use of advances in cellular and molecular mechanisms of signaling pathways to target the TME.

### 4.2. Notch in the Breast TME

Notch regulates many components of the breast TME, such as immune cells but also fibroblasts, endothelial and mesenchymal cells [15]. Notch activation evokes a “Cancer Stem Cell (CSC)” phenotype that results in disease progression, therapy resistance and relapse. In addition, Notch regulates the CSC secretion of paracrine factors and inflammatory cytokines, reshaping the niche, and the niche in turn can support a drug resistance phenotype as well as the modulation of immune response [15]. In this regard, Notch can control the fate of various myeloid cells including macrophages, T-cell types but also CAFs and the ECM, thus resulting in disease progression.

#### 4.2.1. Notch Signaling and CAFs

**Figure 2:**
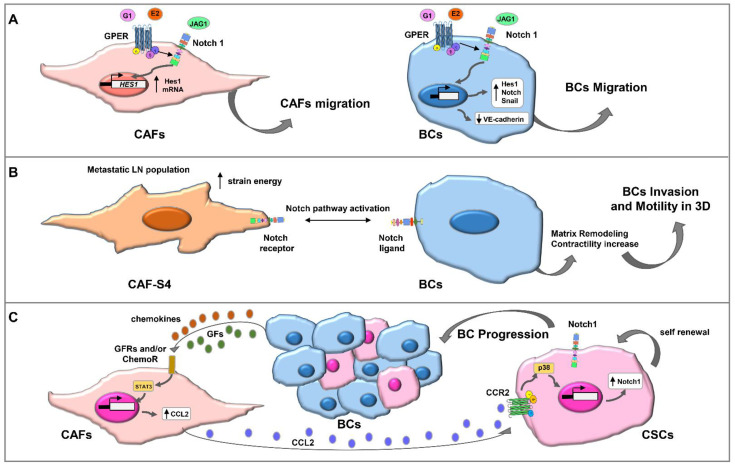
Schematic representation of Notch signaling and CAFs. (**A**) A crosstalk between Notch and GPER signaling pathways occurs in BCs and CAFs. E2 and the GPER selective ligand (G1) induce both the γ-secretase-dependent activation of Notch1 and the expression of the Notch target gene *HES1*. Moreover, E2- and G1-activated GPER triggers Notch-mediated BC and CAF cell migration and *Snail* and *VE-Cadherin* gene expression changes in BCs [80]. (**B**) Several myofibroblast subtypes are highly abundant in invaded LN and correlate with tumor cell invasion. The pro-metastatic CAF-S4 subtype stimulates BC invasion and motility in three dimensions (3D) by increasing contractility and matrix remodeling through Notch pathway activation [81]. (**C**) In TME, paracrine signaling initiated by several BC-secreted GFs and/or chemokines caused an increase in CCL2 mRNA and protein expression levels through STAT3 activation in CAFs; CCL2, in turn, binding to CCR2 at CSCs membrane, induced p38 MAPK phosphorylation followed by Notch1 mRNA and protein expression increases leading to CSCs self-renewal and BC progression [82].

Activation of Notch rather than its loss is implicated in the activation of fibroblasts [15]. Studies indicate the crosstalk between Notch and GPER signaling pathways in CAFs (Figure 2A) and the functional interaction between stroma and BCs extending the potential of the E2 to engage in Notch signaling [80].

Furthermore, a recent elegant study investigated CAFs heterogeneity in metastatic lymph node and tested if one or several CAFs subsets could be involved in BCs spread (Figure 2B) [81]. A subset with contractile properties promotes cancer cell motility and invasiveness through Notch-mediated pathways [81]. Thus, the Notch pathway appears as a prime regulator of tumor cell invasiveness in the tumor-stroma-inflammation setting. CAFs isolated from metastatic BC also express IL-6 which promotes tumor growth and invasiveness through paracrine induction of Notch in cancer cells [83]. In particular, CAFs-secreted CCL2 confers the stem cell phenotype to BCs by activating Notch signaling (Figure 2C) [82]. Therapy resistance pathways are potentially regulated by JAG1/Notch3-mediated crosstalk between CAFs and BC cells, through the expansion of CD44+CD24 low tumor-initiating and therapy-resistant cells [84]. Further findings indicated that Notch signaling mediates the elevated levels of migration and invasion of TNBC cells after their interactions with CAFs in the presence of TNF-α [85]. Additionally, the contact-dependent induction of CXCL8 was Notch dependent. Thus, these findings provide evidence to a novel, Notch-dependent mechanism, which regulates CXCL8 in TNBC, and indicate that this Notch-mediated regulatory mechanism is not shared by all pro-metastatic chemokines, like CCL5 [85].

#### 4.2.2. Notch Signaling and TAMs

**Figure 3:**
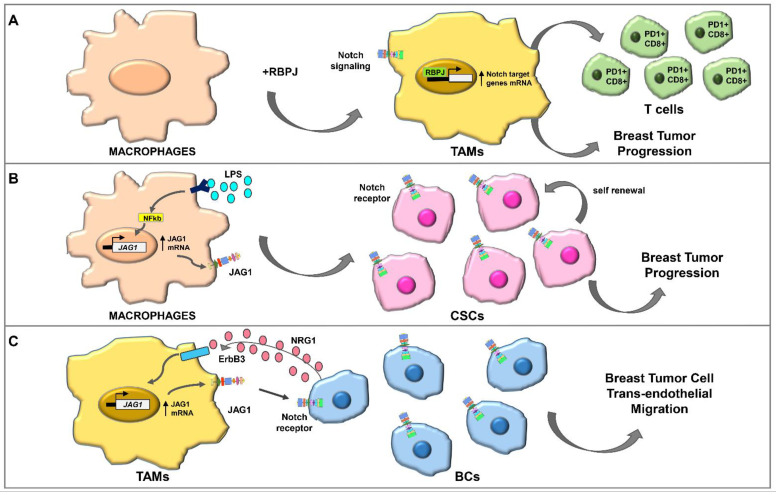
Notch signaling role in breast cancer TME. (**A**) In the macrophages, TAMs’ terminal differentiation is dependent on RBPJ, a transcriptional regulator of Notch signaling; parallel to TAMs expansion, an increase in PD1+ and CD8+ T cells and tumor progression occurs [89]. (**B**) NF-κB activation, induced by LPS, upregulates JAG1 expression in macrophages; this upregulation stimulates Notch signaling in CSCs, leading to an expansion of CSC populations and then to a breast tumor progression [91]. (**C**) A paracrine loop between BCs and TAMs involves NRG1 and Notch signaling; BCs secrete NRG1 that binds ErbB3 receptor in the TAMs, upregulates mRNA and protein expression of JAG1; the latter, in turn, by activating Notch receptor on BCs, stimulates breast tumor cell trans-endothelial migration [92].

Notch receptors and ligands are detected in macrophages including TAMs and influence their behavior in the context of breast TME [86,87]. Macrophage infiltration and Notch ligand expression levels were higher in BC patients resistant to treatment with AI and were significantly associated with poor prognosis [88]. Notch signaling plays a crucial role in TAMs differentiation. The terminal differentiation of macrophages to TAMs depends on RBPJ (Figure 3A) [89,90]; RBPJ-expressing TAMs are consistent with increase of PD1+ CD8+ T-cells and encourage breast tumor burden [89].

Notch favors macrophage differentiation towards a pro-inflammatory phenotype [86,87]. Canonical Notch RBPJ stimulation promotes inflammatory macrophage polarization by determining the expression of IRF8 [93]. Further evidence indicates that in response to pro-inflammatory stimulus, macrophages express Notch ligands in basal-like BC; the authors suggested that these effects could determine Notch activation and proliferation of CSCs and then breast tumor progression (Figure 3B) [91].

Crosstalks between Notch and other macrophages’ signaling represent a fundamental way to promote BC progression [94]. A recent elegant study demonstrates that, in basal-like BC, Notch not only regulates cancer cell expression of the mononuclear cell chemokines IL-1β and CCL2, but also facilitates TGF-β-mediated activation of tumor cells by TAMs, closing a signaling loop between these two cell types [95]. Moreover, Notch influences mitochondrial metabolic reprogramming toward oxidative phosphorylation, further endorsing a pro-inflammatory M1 phenotype [96].

Stimulation of macrophages with ErbB3 ligand NRG1 upregulated canonical ligands of Notch receptor, and this effect by macrophages was important for trans-endothelial migration of tumor cells [92], reinforcing a possible role of Notch/ErbB3 in BC invasion and metastasis (Figure 3C).

However, Notch stimulation can also yield an anti-inflammatory macrophage phenotype together with the expression of specific genes such as *IL-10* and *ARG-1* [97] in response to IL-4 [98]. In BC expression of Notch, canonical ligands favor IL-10-secreting TAMs [88]. In summary, although Notch may regulate interactions between different cells in the TME, the differentiation of Notch-driven macrophages is complex and may be context-dependent.

#### 4.2.3. Notch and the ECM

ECM is crucially involved in the replenishing of growth factors and chemokines that induce a continuous inflammatory state, resulting in expansion of the cellular repertoire. Furthermore, complex ECM remodeling processes are required for cancer cells to invade stromal tissue and create a microenvironment that promotes tumor progression and metastasis [99]. ECM remodeling is influenced by proteinases, MMPs and PA–plasmin systems [100,101]. Plasmin, either directly or indirectly through MMPs, degrades components of the ECM, thus contributing to cancer cell invasion and metastases [102]. It is reported that MMPs are secreted and activated primarily by tumor cells but also by cellular components of the TME [103]. Notch plays a central role in ECM remodeling by regulating the expression of proteinases [29]. In TNBC, Notch1 increases the expression of MMP-2 and MMP-9, through NF-κB signaling [104]. Expression data in both BC cell lines and primary tumors demonstrate an association between elevated expression of canonical Notch ligands such as JAG1, the uPA and the basal-like BC subtype. The uPA promoter contains a CBF-1 binding site required for direct transcriptional regulation by Notch [105]. These data suggest that ligand-induced Notch activation results in BC progression through upregulation of the uPA and these findings link these pathways and the poor prognosis. In a follow-up study, the Notch-regulated uPA–plasmin axis activates latent TGF-β in stroma cells [106,107], which stimulates the TGF-β receptor 1, another Notch target gene in BCs [95]. Several reports have recently revealed that the ECM proteins Periostin and Tenascin C play key roles as metastasis niche components for tumor-initiating cells that invade different organs [108,109]. By enhancing Wnt and Notch signaling in cancer cells, these proteins provide physical as well as signaling support for metastasis-initiating cells [110].

#### 4.2.4. Notch Signaling and Angiogenesis

Several molecular mechanisms are involved in core cellular metabolism reprogramming, which is an important feature of neoplastic cells [111,112,113]. During fast BC proliferation, a profuse demand for nutrients and oxygen as well as the rapid depletion of metabolic waste from the TME are required. Thus, a dense microvasculature develops due to pathological tumor angiogenesis and allows the intense blood–tissue exchange to deliver oxygen and nutrients for tumor development. Tumor microvessels are characterized by hyperpermeable endothelium, which permits intense transmigration of inflammatory cells into tumor and cancer cell dissemination, but also the accumulation of different molecular agents shaping the TME [112].

Aberrant Notch signaling is involved in different mechanisms regulating cancer angiogenesis. More recently, the Notch pathway is emerging as a novel mediator of angiogenesis and vasculogenesis [57]. JAG1, expressed in tumor cells, has been shown to activate Notch signaling in neighboring endothelial cells to promote angiogenesis [114]. Notch can also regulate angiogenesis by limiting the number of tip cells formed and by promoting arterial cell fate [115].

However, angiogenesis is a multifactorial process in which cell–cell communication is fundamental through actions of multiple juxtacrine pathways, adhesion molecules and ECM [116]. The most characterized mechanism is dependent on the VEGF, highly expressed in different types of cancer including breast [117]. Interestingly, it is reported that the complex interplay between Notch and VEGF signaling regulates the source of the ligands and receptors of these pathways during angiogenesis [57]. Although the Notch pathway regulates the expression of VEGF receptors, it is also reported that VEGF coordinates tumor endothelial expression of Dll4, which serves as a negative feedback regulator of vascular growth [57,118]. JAG1 alters the balance between Dll4-Notch and VEGFRs by antagonizing Dll4-mediated Notch activation. This reduction allows tip and stalk cells to change positions, resulting in a dense and tortuous vascular network [119,120]. JAG1 also induces the expression of VEGFR3, which influences the expression of both pro- and anti-angiogenic factors [121]. Contact between macrophages and endothelial cells allows for Notch-mediated induction of sprouting angiogenesis [119]. It was further demonstrated that in BC, Notch ligand/Notch3 is highly expressed in blood vessels and implicated in tumor angiogenesis [95].

## 5. Notch Signaling in the Emergence of Drug Resistance Dependent on TME

The clinical management of BC has improved in the last twenty years, although therapeutic resistance is still a challenge [122]. Breast tumors develop drug resistance through a variety of mechanisms, with the TME serving pivotal roles [123]. Indeed, responses of BCs to different drugs are crucially influenced by deriving signals from the TME. Numerous studies demonstrate that during different drugs’ stimulation, the TME contributes by controlling the behavior of cancer cells [124], even though concomitantly the different cellular components such as TAMs, receive paracrine factors from BCs and this bidirectional crosstalk contributes to the acquired drug resistance [125]. For instance, inhibition of CSF-1 expressed by cancer cells, which is implicated in engaging macrophage in tumors, could reverse chemoresistance in BCs [126]. Furthermore, it has emerged that the effects of different drugs on macrophage phenotype may vary, due to breast heterogeneity, assuming breast tumor-specific evaluation of the potential therapeutic effects of macrophage modulation.

The cellular heterogeneity of the TME, the abnormal Notch pathway, and the activation of other signals [15] are retained crucial determinants among the probable mechanisms underlying therapeutic resistance in BC [56]. Although this issue needs further insights, Notch signaling emerges as a major component that contributes to drug resistance at any specific stage of tumorigenesis in BC patients [56].

Notch signaling is upregulated in endocrine-resistant BCs and is involved in modulating TAMs’ differentiation (Table 2).

The JAG1-Notch pathway regulates TAM polarization and promotes the acquisition of AI resistance by upregulating TAM infiltration [88].

TAMs can collaborate with CAFs via cell–cell interaction to promote endocrine resistance and Notch signaling may contribute in the crosstalk between these two cell types. Different CAF phenotypes influence diverse responses to drugs in BCs [133]. Particularly, crosstalk among Fibronectin, fibroblast-derived factor and its receptor [134] has been described as a molecular mechanism for endocrine resistance. Fibroblasts have also been shown to promote therapy resistance in BCs through expression of Jagged1 and exosomal transfer, leading to activation of Notch3 and STAT1 signaling [84]. Thus, targeting Notch signaling in CAFs and TAMs could be a promising therapeutic strategy to improve clinical outcome for endocrine-resistant BC patients.

CSCs may be responsible for treatment resistance, through self-renewal capacity and differentiation [135]. Self-renewal of CD133 high expressing cells by IL-6/Notch3 signaling regulates endocrine-resistance in metastatic BC [127]. Moreover, recent data indicate that one consequence of endocrine therapy is the activation of Notch to promote survival of breast CSCs and resistance to endocrine therapy [56,136]. Mutations that constitutively activate ERα without hormone binding are frequently found in endocrine-therapy-resistant BC metastases and are associated with poor patient outcomes. Specifically, the Y537S mutation results in a constitutively active ER, thus causing endocrine therapy resistance in ER+ BC [137]. MCF-7 cells expressing mutant (Y537S) ERα show increased mammosphere-forming efficiency, compared to wild-type ERα-expressing cells, together with enhanced expression of Notch4, Notch4 intracellular region Notch4IC, JAG1, Dll1, and Dll3 [128].

Further evidence indicates that progression of ER+ BC depends on Dll1-mediated Notch signaling and its effects on CSCs [138]. However, it is not yet clear whether the tumor-promoting function of Dll1 on CSCs is also responsible for endocrine resistance. Doxorubicin or docetaxel, as well as anti-estrogens, such as tamoxifen or fulvestrant, result in an enrichment of ALDH+ breast CSCs populations, resistant to these therapies [56]. A study demonstrated that short-term treatment with tamoxifen or fulvestrant inhibits proliferation of ALDH/ER+ BCs but increases survival and self-renewal of ALDH+/ER− BCSCs through JAG1-Notch4 receptor activation, in both patient-derived samples and xenograft tumors. In fact, the use of RO4929097, a Notch4 inhibitor, reverses the anti-estrogen-dependent Notch and BCSC activity increase [129]. Moreover, an increase in Notch target genes (*HEY1* and *HES1*) expression was observed in ALDH+/ER− BCSCs [129]; these results indicate that BCSCs and *Notch4*/*HEY*/*HES* gene signature predict acquired tamoxifen resistance and suggest that endocrine therapy in combination with JAG1/Notch4 inhibitors could overcome resistance in BC. More recently, Shah et al. [130] indicate that higher membrane JAG1 expression may be used to either predict response to anti-HER2 therapy or for the detection of Notch-sensitive CSCs post therapy. Sequential blockade of HER2 followed by JAG1 or Notch could be more effective than simultaneous blockade to prevent drug resistance and tumor progression [130]. In another study, it has been demonstrated that Notch1 contributes to trastuzumab resistance by repressing PTEN that leads to hyperactivation of ERK1/2; this may promote HER2+ BC cell proliferation and stem cell survival [131]. Furthermore, a recent study reports that Notch signaling is augmented in endocrine-resistant BCs following a global reprogramming of the epigenome [139].

The maintenance of stemness and hypoxic TME contribute to BC drug resistance. A study demonstrated that chronic hypoxia induces BC cell resistance to paclitaxel and promotes stem phenotype conversion through high expression of HIF-2α by activating Wnt and Notch pathways [132]. These findings highlight the importance of a new mechanism involving Notch by connecting resistance and the TME in BC [132]. Additionally, Notch signaling activation strongly correlates with the invasiveness and chemoresistance of TNBC [13]. An in vitro study revealed that paclitaxel’s cytotoxic effect was enhanced after Notch1 silencing by upregulating Caspase-3 and Caspase-9 expression and inhibiting Bcl-2 in MDA-MB-231 TNBC cells [140]. Chemoresistance of BC-related bone metastasis was also evaluated. It is reported that targeting osteoblastic JAG1 induced by chemotherapy could improve response to chemotherapy in TNBC patients, who develop bone metastasis. The human monoclonal antibody against JAG1 synergizes with chemotherapy to reduce bone metastasis and dramatically reduces metastatic relapse to bone from primary tumors [141].

## 6. Conclusions

It is commonly accepted that inappropriate Notch signaling can occur in BC through upregulation of Notch receptors, ligands and co-activators or the loss of transcriptional co-repressors. The pleiotropic effects of Notch are the result of the transcriptional cascade triggers that culminate in specific target genes’ activation and/or repression, as well as in epigenetic transcriptional modulation. Well-characterized target genes have been described, including those belonging to the *HES* and *HEY* gene family, *CCND1* and *MYC*, influencing cancer cell proliferation. Evidence also indicates that Notch crosstalks with other oncogenic pathways involved in BC carcinogenesis, such as those mediated by developmental signals (e.g., Wnt), growth factors (e.g., VEGFR), cytokines, receptors (e.g., ER, HER2) and oncogenic kinases (e.g., MAPK).

Furthermore, recent studies have evidenced that Notch signaling plays an important role in regulating the complex interaction among cells composing the TME which are important for determining BC progression and drug resistance. Many features of TME, including mesenchyme interaction and activation, various functions of the immune infiltrate and vascular architecture, are regulated by Notch through juxtacrine and paracrine signaling. Notch promotes fibroblast activation in CAFs and it is implicated in the crosstalk of pro-metastatic CAFs and BCs that regulate BC cancer cell motility and invasiveness. Moreover, in the TME, paracrine signaling by BCs stimulates CAFs to secrete specific cytokines which are involved in Notch-dependent CSCs’ self-renewal and BC progression. Regulation of immunosuppressive environment components is also under Notch activity’s control such as TAM differentiation. Furthermore, pro-inflammatory conditions can stimulate Notch ligand expression in the macrophages leading to Notch-dependent CSCs’ self-renewal and then BC progression. This latter event is also influenced by a crosstalk between Notch and macrophage signaling. Notably, Notch signaling emerges as a determinant for other characteristic aspects of tumor progression, including ECM remodeling, EMT promotion, angiogenesis, vasculogenesis and cancer stem cell maintenance. In fact, the enrichment of BCSCs within the bulk tumor is dependent on Notch signaling deregulation. Notch-overexpressing CSCs have self-renewing properties, drug resistance and more aggressiveness.

These recent findings support the targeting of Notch signaling interactions between cancer cells, TME and CSCs as a new therapeutic strategy against BC. However, further studies are necessary to understand the mechanisms of acquired chemoresistance through Notch-dependent tumor−stromal interactions and to find predictive biomarkers for response to such TME-directed chemoresistance therapies.

## Figures and Tables

**Table 1:** Significance of Notch signaling dysregulation in BC. (↑: increase).

Notch Receptors or Ligands	Experimental Models	Evidences	References
Notch1 ↑ Notch3 ↑	MMTV/Notch1 transgenic mice MMTV/Notch3 transgenic mice	Mammary gland tumor formation	[39]
Notch1 ↑	Normal and tumorigenic human mammary epithelial cell lines	Breast epithelial cells transformation Cell proliferation ↑ Apoptosis suppression	[41]
Notch4 ↑	MDA-MB-231 cells BC xenograft models	Cell proliferation and invasiveness ↑ Apoptosis suppression Xenografts tumor growth ↑	[42]
Notch1 Notch4	ERα+ and ERα- BC cells ERα- BC xenograft models	E2/ ERα inhibition of Notch ERα- BC cells proliferation ↑ Xenografts tumor growth ↑	[43]
Notch1	ErbB2 normal and ErbB2 overexpressing human DCIS cell lines BC patient samples	Notch and ErbB1/2 crosstalk regulates DCIS acini size and mammosphere formation	[44]
Notch1 ↑	ERα+ and ERα- BC cells BC xenograft models BC patient samples	Notch1 and Ras/MAPK crosstalk regulates BCs spheres formation and xenografts tumor growth	[45]
JAG1/Notch1 ↑	MDA-MB-231 cells BC xenograft models	EMT Anoikis inhibition Xenografts tumor growth and metastasis ↑	[46]
Dll1 ↑	MCF-7, BT474 cells	Proliferation, migration, and invasion ↑ Apoptosis suppression	[47]

**Table 2:** Significance of Notch pathway in TME-dependent drug resistance. (↑: increase; ↓: decrease).

Drugs	Targeted Notch Receptors or Ligands	Experimental Models	Evidences	References
Anastrozole, Letrozole, Exemestane	JAG1	AI resistant BC cells AI resistant BC patient samples	M2 TAM proportion ↑	[88]
Tamoxifen, Fulvestrant	IL6/Notch3 signaling activation	Hormonal therapy resistant cells In vivo xenograft BC models	CD133 high/ ER low/IL6 high CSCs self-renewal ↑	[127]
Tamoxifen	Notch 4 ↑ JAG1 ↑ Dll1-3 ↑	MCF7 Y537S-ERα cells	Mammosphere-forming efficiency ↑ Endocrine resistance	[128]
Tamoxifen, Fulvestrant,	JAG1/Notch4 activation	ALDH+/ER− BCSCs patient-derived cells In vivo patient-derived xenograft BC models	BCSCs self-renewal ↑	[129]
Lapatinib	JAG1 ↑ Notch1/3/4 ↑	HER2 overexpressing BC cells	CSCs enrichment and tumor initiation	[130]
Trastuzumab	Notch1 ↑	Trastuzumab resistant HER2+ BC cells	PTEN ↓ ERK1/2 ↑ BCSCs survival and self-renewal ↑	[131]
Paclitaxel	Notch signaling activation	ER+ and TNBC cells Xenograft BC models	HIF2α ↑ Stem phenotype	[132]

## Data Availability

Not applicable.

## Abbreviations

AI	aromatase inhibitors
ALDH+	aldehyde dehydrogenase positive
ARG-1	arginase-1
BC	breast cancer
BCs	breast cancer cells
BCSCs	breast cancer stem cells
bHLH	Hes/Hey family of basic helix–loop–helix
CAFs	cancer-associated fibroblasts
CBF-1	centromere binding factor 1
CCL17	C-C motif chemokine ligand 17
CCL2	C-C motif chemokine ligand 2
CCL22	C-C motif chemokine ligand 22
CCND1	cyclin D1
CCR2	C-C chemokine receptor type 2
CDKI	cyclin-dependent kinase inhibitor
ChemoR	chemokines receptors
CSCs	cancer stem-like cells
CSF-1	colony stimulating factor-1
DCIS	ductal carcinoma in situ
E2	estradiol
ECM	extracellular matrix
EGF	epidermal growth factor
EMT	epithelial mesenchymal transition
ER	estrogen receptor
ER+	estrogen receptor alpha positive
ER−	estrogen receptor alpha negative
ErbB1	human epidermal growth factor receptor
ErbB2	human epidermal growth factor receptor 2
ErbB3	human epidermal growth factor receptor 3
ERα	estrogen receptor alpha
GFRs	growth factor receptors
GFs	growth factors
GPER	G protein-coupled estrogen receptor 1
GSIs	γ-secretase inhibitors
GSK3-β	glycogen synthase kinase 3 beta
HER2	human epidermal growth factor receptor 2
HER2+	human epidermal growth factor receptor 2 positive
HIF-1α	hypoxia-inducible factor 1 alpha
HIF-2α	hypoxia-inducible factor 2 alpha
IL-10	interleukin-10
IL-1β	interleukin-1 beta
IL-4	interleukin-4
IL-6	interleukin-6
IRF8	interferon regulatory factor 8
JAG	membrane-bound ligands Jagged
LN	lymph nodes
LPS	lipopolysaccharide
MAML	mastermind-like
MAPK	mitogen-activated protein kinase
MMP-2	matrix metalloproteinase-2
MMP-9	matrix metalloproteinase-9
MMPs	matrix metalloproteinases
MMTV	mouse mammary tumor virus
MSCs	mesenchymal cells
NF-κB	nuclear factor kappa B
NECD	Notch extracellular domain
NICD	Notch intracellular domain
NRG1	neuregulin 1
Oct-4	octamer binding transcription factor 4
PA	plasminogen activator
PD1+	programmed death 1 positive
PR	progesterone receptor
PR+	progesterone receptor positive
PTEN	phosphatase and tensin homolog
RBPJ	recombination signal binding protein for immunoglobulin kappa J region
SDF-1	stromal cell-derived factor-1
STAT1	signal transducer and activator of transcription 1
TAMs	tumor-associated macrophages
TGF-β	transforming growth factor beta
TILs	tumor-infiltrating lymphocytes
TME	tumor microenvironment
TNBCs	triple negative breast cancers
TNF-α	tumor necrosis factor alpha
uPA	urokinase-type plasminogen activator
VEGF	vascular endothelial growth factor
VEGFR3	VEGF receptor 3
VEGFRs	vascular endothelial growth factor receptors
